# Characterization of clinicopathological features, treatment practices, and outcomes among Finnish advanced breast cancer patients in real-life clinical practice

**DOI:** 10.1007/s00432-023-04723-0

**Published:** 2023-05-13

**Authors:** Krista Heinolainen, Silva Saarinen, Simona Vertuani, Antti Ellonen, Antti Karlsson, Meri Utriainen, Peter Carlqvist, Jami Mandelin, Barbro Holm

**Affiliations:** 1 Novartis Finland Oy, Espoo, Finland; 2 Novartis Sverige AB, Kista, Sweden; 3 Department of Oncology, Turku University Central Hospital, Turku, Finland; 4 Auria Biobank and University of Turku, Turku, Finland; 5 Comprehensive Cancer Center, Helsinki University Central Hospital, Helsinki, Finland; 6 Nordic Market Access, Stockholm, Sweden; 7 Faron Pharmaceuticals, Turku, Finland

**Keywords:** Advanced breast cancer, PIK3CA mutation screening, Retrospective register study, Breast cancer subtypes, Breast cancer treatment

## Abstract

**Purpose:**

In recent years, several new targeted therapies have emerged for advanced breast cancer (aBC). However, real-life data specific to aBC and different breast cancer subtypes are scarce. This retrospective cohort study was designed to describe the distribution of aBC subtypes, incidence, treatment patterns, survival, and *PIK3CA* hotspot mutation frequency.

**Methods:**

The study included all patients in the Hospital District of Southwest Finland diagnosed with aBC between 2004 and 2013 and with a sample available in Auria Biobank. In addition to registry-based data collection, 161 HR+/HER2− aBCs were screened for *PIK3CA* mutations.

**Results:**

Altogether, 54.7% of the 444 patients included in the study had luminal B subtype. The smallest representations were in HR−/HER2+ (4.5%) and triple-negative (5.6%) subgroups. The percentage of aBC among all diagnosed breast cancers increased until 2010, after which it remained stable. The triple-negative cancers were associated with shorter median overall survival (5.5 months) compared to other subgroups (16.5–24.6 months). Most (84%) triple-negative cancers also metastasized during the first two years, whereas this was more evenly distributed over time in other subgroups. Of the HR+/HER2− tumors, 32.3% harbored a *PIK3CA* hotspot mutation. These patients, however, did not have inferior survival compared to patients with *PIK3CA* wild-type cancers.

**Conclusion:**

This study described real-world aBC subgroups and indicated that the clinical outcomes of subgroups vary. Although *PIK3CA* hotspot mutations did not lead to inferior survival, they are relevant as possible treatment targets. Overall, these data could be utilized to further evaluate the subgroup-specific medical needs in breast cancer.

**Supplementary Information:**

The online version contains supplementary material available at 10.1007/s00432-023-04723-0.

## Introduction

Female breast cancer (BC) is the most common cancer and one of the leading causes of cancer-related deaths worldwide (Sung et al. 2021). Finland is one of the countries with the highest incidence rates of BC, but the prognosis is among the best in Europe (Sung et al. 2021; Suomen Rintasyöpäryhmä RY 2023). In 2019, slightly over 5000 BC cases and nearly 900 BC-related deaths were reported (Pitkäniemi et al. 2019). The 5-year relative survival rate was as high as 91% (Pitkäniemi et al. 2019).

More than 90% of patients with BC are diagnosed with early-stage disease. Only around 5% of newly diagnosed patients present BC with distant metastasis (i.e., de novo metastatic disease), and in 15−20% of treated early-stage BC patients the cancer reappears later as an inoperable locally advanced or metastatic disease (i.e., advanced breast cancer, aBC) (Mattson and Huovinen 2015; Cardoso et al. 2020). In patients with aBC, the median overall survival (mOS) is around three years [4].

Breast cancer is a heterogeneous disease that can be divided into subgroups using different classification methods. These subgroups are associated with different therapeutic response patterns and clinical outcomes (Yersal and Barutca 2014). Biologically, BC is classified into four molecular subtypes: luminal A, luminal B, human epidermal growth factor receptor 2 positive (HER2+), and basal-like (also called triple-negative, as they do not express estrogen receptors (ER), progesterone receptors (PR), and HER2) (The Cancer Genome Atlas Network 2012). This classification is based on comprehensive gene expression profiling that is currently not widely used in the clinical setting (Suomen Rintasyöpäryhmä RY 2023). However, a similar classification based on four markers, ER and PR status (together denoted as hormone receptor, HR status), HER2 oncogene expression, and proliferation rate (Ki67), is used in clinical practice. Determining the status of these four markers is an essential step in the treatment algorithm of BC, as it guides treatment decisions related to hormonal and HER2-targeted therapy.

The development of the Next Generation Sequencing (NGS) technology has enabled wide characterization of genes involved in cancer progression (The Cancer Genome Atlas Network 2012). The most frequently mutated driver genes in BC are *Phosphatidylinositol-4,5-Bisphosphate 3-Kinase Catalytic Subunit Alpha* (*PIK3CA,* coding for the α subunit of PI3K) in luminal A subtype (45%), *PIK3CA* and *Tumor Protein P53* (*TP53)* in luminal B (29% each), *TP53* in basal-like (80%), and *TP53* and *PIK3CA* in HER2+ cancers (72% and 39%, respectively). Up to 80% of *PIK3CA* mutations occur in mutational hotspots affecting the helical (E542K and E545K) and kinase (H1047R) domains of the corresponding catalytic subunit alpha protein (p110α). These, among other driver genes, also have clinical significance in cancer classification and treatment. The recently revised European Society for Medical Oncology (ESMO) guidelines recommend assessing *PIK3CA* mutation status in HR+/HER2− metastatic BC as part of routine clinical practice (Gennari et al. 2021).

The Finnish Breast Cancer Group maintains the National Guidelines for Breast Cancer Diagnostics and Treatment in Finland (Suomen Rintasyöpäryhmä RY 2023). BC treatment is tailored according to tumor subtype and stage, patient’s status, and previous treatments (Mattson and Huovinen 2015). In recent years, several new targeted therapies have been introduced or are currently under investigation for aBC, such as cyclin-dependent kinase 4 and 6 (CDK4/6) and PI3K inhibitors for the treatment of HR+/HER2− aBC (Gennari et al. 2021). The first-in-class α-specific PI3K inhibitor alpelisib was approved by EMA in 2020 and is indicated in combination with fulvestrant for the treatment of postmenopausal women, and men, with an HR+/HER2− aBC with *PIK3CA* mutation after disease progression following endocrine monotherapy (André et al. 2019, 2021).

As multiple novel treatments are indicated for the specific subgroup of patients with aBC, there is a need for a better understanding of the epidemiology, clinicopathological characteristics, and current treatment patterns of aBC, at a subtype-specific level. Much is known about general BC epidemiology in Finland, mainly owing to the Finnish Cancer Registry. Data specific to aBC and different BC subtypes, including mutational frequencies of *PIK3CA*, however, is currently scarce.

The primary objective of this study was to describe the distribution of different aBC subtypes in the Hospital District of Southwest Finland between 2004 and 2013. The secondary objectives were to describe clinical characteristics, treatment patterns, and survival in patients with aBC, and to estimate aBC incidence in Southwest Finland. In addition, the frequency of *PIK3CA* hotspot mutations were evaluated in a sub−cohort of HR+/HER2− aBC.

## Materials and methods

This study was a non-interventional, retrospective registry-based cohort study. The study cohort included all patients in the Hospital District of Southwest Finland who met the following inclusion criteria: diagnosed with aBC (tumor stage IIIb–IV) during January 1, 2004–August 31, 2013, aged ≥ 18 years at the time of aBC diagnosis, and with a sample available in Auria Biobank. Patients were followed-up until death or December 31, 2016, whichever occurred first. Data were retrospectively collected from the Auria Biobank database, electronic medical records (EMR) from Turku University Central Hospital, Drug Reimbursement Registry (Finnish Social Insurance Institution, Kela), and Cause of Death Registry (Statistics Finland). The validation of the subject population was done in several steps, including keyword-based text algorithms to identify aBC patients and to confirm their tumor staging from EMRs manually.

The following variables were collected: sex, menopausal status, age at aBC diagnosis, type of aBC (de novo*,* incl. metastasis < 6 months of the initial diagnosis, or recurrent), number of metastatic sites, tumor marker profile (ER/PR, HER2, and Ki67), comorbidities, number and type of aBC treatment regimens, date of initial BC and aBC diagnosis, and date of death. Additionally, the number of new BC diagnoses per year in Southwest Finland between 2004 and 2013 were collected.

The date of aBC diagnosis and date of death were used to estimate overall survival (OS). Metastasis-free survival (MFS) was defined as the time from the initial early BC diagnosis to aBC diagnosis (with local or distant metastasis).

In addition, formalin-fixed, paraffin-embedded (FFPE) tumor tissue samples of all patients with HR+/HER2− aBC were collected from Auria Biobank’s tissue archives and screened for *PIK3CA* hotspot mutations (C420R, E542K, E545x, Q546x, and H1047x).

### Patient subgroups

Three different classification methods were used to provide data on different levels of granularity. Classification into five BC subtypes (classification 1) was done based on the tumor marker profile at the time of diagnosis: luminal A (ER+, PR+, HER2−, Ki67 low) (total mean follow-up: 278.5 months), luminal B (ER+, PR−, HER2−, Ki67± ; or ER+, PR+, HER2−, Ki67+ ; or ER+ , PR± , HER2+ , Ki67±) (642.2 months), triple-negative (ER−, PR−, HER2−) (21.2 months), HR−/HER2+ (ER−, PR−, HER2+) (39.0 months), and subtype unknown (153.9 months). In the second classification (classification 2), patients were divided into two groups: HR+/HER2− (689.9 months) and not-HR+/HER2− (including patients with HR and/or HER2 status unknown) (445.0 months). In the third classification (classification 3), patients were divided by HER2 status (HER2+ or not-HER2+/status unknown) (253.6 and 881.3 months, respectively). According to the clinical practice during the study period, ER/PR positivity was defined using a threshold of ≥ 1%, and HER2 positivity by immunohistochemistry score 3+ or positive in situ hybridization result.

Additionally, the archived tumor tissue samples of patients with HR+/HER2− aBC were screened for *PIK3CA* hotspot mutations and, based on the results, these patients were further classified into three additional subgroups: cases with the above-mentioned *PIK3CA* hotspot mutation (hotspot), *PIK3CA* mutations outside hotspots (other), and no *PIK3CA* mutation detected (wild-type, wt) (see, *PIK3CA* mutation screening).

### Treatment patterns

Data on hospital-administered medication and anti-cancer drug prescriptions were used to determine treatment lines (treatment regimen and the length of the treatment line). The treatment line was defined by the pharmacological agent or combination of agents administered. For further analyses, the treatment regimens were subcategorized to anti-HER2, anti-HER2+ chemotherapy, anti-HER2+ endocrine therapy, chemotherapy only, endocrine therapy only, or other therapy.

### PIK3CA mutation screening

Genomic DNA was extracted from the FFPE tumor samples. The NGS gene panel QIAseq Human Actionable Solid Tumor Panel (QIAGEN, Hilden, Germany) was used for mutation screening and it included the whole coding region of *PIK3CA* p110α. The library preparation was performed according to the manufacturer’s instructions (QIAGEN). Sequencing was performed with Illumina NextSeq 500 (Illumina Inc., San Diego, CA) in the Pathology unit at Central Finland Central Hospital (Jyväskylä, Finland).

Data processing was performed with CLC Genomic workbench software (QIAGEN) and annotation and interpretation using omnomics NGS software (Euformatics, Espoo, Finland). Detection of mutations included point mutations, small insertions, and small deletions.

### Statistical analyses

Descriptive analyses were conducted to assess demographic characteristics, treatment patterns, and MFS. Distributions of continuous variables were expressed as mean with standard deviation (SD), median, and range, and categorical variables as number and percentage of proportions. Only existing data were utilized, and no missing values were imputed. The Kaplan–Meier estimate was used to estimate OS.

Python v3.6 (www.python.org) was used to enhance data collection from the Auria Biobank database and EMRs, and to perform descriptive statistics. Survival analyses were performed with SPSS Statistics software v26 (https://www.ibm.com/analytics/data-science/predictive-analytics/spss-statistical-software).

### Ethical considerations

The study approval was obtained from the Scientific Steering Committee of Auria Biobank (AB17-3826) and Hospital District of Southwest Finland (T147_208). The study was performed in accordance with the declaration of Helsinki and in compliance with applicable national laws. The retrospectively collected tissue samples and related clinical data have been transferred to the Biobank by public announcement and can be used for scientific research purposes without informed consent according to the Finnish Biobank Act (688/2012).

## Results

### Clinicopathological features

A total of 444 aBC patients were included in the study. The mean age at diagnosis was 66.0 years (SD 13.8; range 20.6−102.5) (Table 1). The percentage of aBC cases of all diagnosed BC increased until 2010, after which it remained stable. The highest incidence of 61 cases was seen in 2010 when 11.9% of diagnosed BC cases were advanced (Table 2).

**Table 1:** Patient characteristics and distribution of different aBC subtypes.

Characteristics	*N* (%)
**All patients**	444 (100)
**Gender**	
*Female*	444 (100)
**Type of aBC**	
*De novo*	165 (37.2)
*Recurrent*	279 (62.8)
**Age group at aBC diagnosis**	
*≤ 20*	0 (0)
*(20, 30]*	2 (0.5)
* (30, 40]*	12 (2.7)
*(40, 50]*	40 (9.0)
* (50, 60]*	97 (21.8)
* (60, 70]*	113 (25.5)
* (70, 80]*	105 (23.6)
*(80, 90]*	65 (14.6)
* > 90*	10 (2.3)
**Mean age (y) at aBC diagnosis (range)**	66.0 (20.6–102.5)
**Menopausal status**	
*Postmenopausal*	390 (87.8)
**Breast cancer subtypes**	
Classification 1	
*Luminal A*	97 (21.8)
*Luminal B*	243 (54.7)
*Triple-negative*	25 (5.6)
*HR−/HER2+*	20 (4.5)
*Unknown*	59 (13.3)
Classification 2	
*HR+/HER2−*	288 (64.9)
*Not-HR+/HER2−/unknown*	156 (35.1)
Classification 3	
*HER2+*	84 (18.9)
*Not-HER2+/unknown*	360 (81.1)
**Number of metastatic sites at diagnosis**	
*1*	228 (51.4)
*2*	120 (27.0)
*3*	58 (13.1)
*4*	22 (5.0)
*5*	12 (2.7)
*6*	4 (0.9)

*aBC* advanced breast cancer, *HER2* human epidermal growth factor receptor 2, *HR* hormone receptor

**Table 2:** The number of new breast cancer and aBC (advanced breast cancer) diagnoses per year and the percentage of aBC in January 1, 2005–August 31, 2013

Year	Breast cancer	aBC	% aBC
2005	714	41	5.7
2006	589	45	7.6
2007	510	41	8.0
2008	537	47	8.8
2009	518	46	8.9
2010	511	61	11.9
2011	504	52	10.3
2012	573	59	10.3
2013 (until August)	317	32	10.1

Patients were classified into BC subtypes based on their tumor marker profile. Three different classification methods were used. The distribution of aBC subtypes is presented in Table 1. According to classification 1, most patients belonged to the luminal B subgroup (54.7%; 243/444). The smallest representations were in the HR−/HER2+ (4.5%; 20/444) and the triple-negative subgroups (5.6%; 25/444) (Table 1).

At the time of diagnosis, 51.4% of the aBC patients (228/444) had a metastasis at one site, and 8.6% (38/444) at four to six metastatic sites (Table 1). The most common comorbidities were hypertension (18.7%) and diabetes (7.2%).

### Treatment patterns

Most aBC patients (387/444, 87.2%) received first-line therapy and 65.1% (289/444) also second-line therapy. Most first-line treatments among all aBC patients included endocrine therapy only (41.6%) or chemotherapy only (40.8%) (Supplementary Table 1). These were also the most common second- and subsequent-line therapies. Anti-HER2 therapy combined with chemotherapy was received by 13.2% of the aBC patients as first-line and by 6.6% as second-line therapy (Supplementary Table 1). The mean number of treatment lines per patient was 3.3 (SD 2.3; range 1–12), and the mean duration of first-line therapy was 9.3 months, second-line therapy 14.5 months, and 6.4 months in later lines.

Examining therapy lines by BC subgroups, the percentage of patients who had received first-line therapy varied from 72.0% (triple-negative) to 90.7% (luminal A) in classification 1. Similarly, 90.3% of HR+/HER2− patients (classification 2) and 85.7% of HER2 + patients (classification 3) had received first-line therapy (Supplementary Table 2). The most common first-line treatment was endocrine therapy only for luminal A (56.8%); chemotherapy only for luminal B (40.6%) and triple-negative (100.0%); anti-HER2 therapy combined with chemotherapy for HR−/HER2+ (80.0%); chemotherapy only for HR+ /HER2− (48.8%); and anti-HER2 therapy combined with chemotherapy for HER2+ (68.1%) (Table 3).

**Table 3:** Distribution of treatment types in different therapy lines by aBC subgroup. Groups of less than five patients were not included in the analysis

Therapy line	Subgroup	No. of observed lines	Anti-HER2 therapy n (%)	Anti-HER2+ endocrine n (%)	Anti-HER2+ chemo-therapy n (%)	Chemo-therapy only n (%)	Endocrine only n (%)	Other treatment n (%)
**First line**	Classification method 1							
	*Luminal A*	88	< 5 (−)	< 5 (−)	< 5 (−)	36 (40.9)	50 (56.8)	< 5 (−)
	*Luminal B*	219	< 5 (−)	< 5 (−)	38 (17.4)	89 (40.6)	83 (37.9)	5 (2.3)
	*Triple-negative*	18	< 5 (−)	< 5 (−)	< 5 (−)	18 (100.0)	< 5 (−)	< 5 (−)
	*HR−/HER2+*	15	< 5 (−)	< 5 (−)	12 (80.0)	< 5 (−)	< 5 (−)	< 5 (−)
	*Unknown*	47	< 5 (−)	< 5 (−)	< 5 (−)	14 (29.8)	23 (48.9)	< 5 (−)
	Classification method 2							
	*HR+/HER2−*	260	< 5 (−)	< 5 (−)	< 5 (−)	127 (48.8)	120 (46.2)	11 (4.2)
	*Not-HR+/HER2− or unknown*	127	< 5 (−)	< 5 (−)	< 5 (−)	31 (24.4)	41 (32.3)	< 5 (−)
	Classification method 3							
	*HER2+*	72	< 5 (−)	< 5 (−)	49 (68.1)	6 (8.3)	13 (18.1)	< 5 (−)
	*Not-HER2+ or unknown*	315	< 5 (−)	< 5 (−)	< 5 (−)	152 (48.3)	148 (47.0)	< 5 (−)
**Second line**	Classification method 1							
	*Luminal A*	65	< 5 (−)	< 5 (−)	< 5 (−)	21 (32.3)	38 (58.5)	6 (9.2)
	*Luminal B*	166	9 (5.4)	12 (7.2)	14 (8.4)	37 (22.3)	83 (50.0)	11 (6.6)
	*Triple-negative*	11	< 5 (−)	< 5 (−)	< 5 (−)	11 (100.0)	< 5 (−)	< 5 (−)
	*HR-/HER2+*	12	7 (58.3)	< 5 (−)	5 (41.7)	< 5 (−)	< 5 (−)	< 5 (−)
	*Unknown*	35	< 5 (−)	< 5 (−)	< 5 (−)	10 (28.6)	23 (65.7)	< 5 (−)
	Classification method 2							
	*HR+/HER2−*	191	< 5 (−)	< 5 (−)	< 5 (−)	56 (29.3)	118 (61.8)	15 (7.9)
	*Not-HR+/HER2− or unknown*	98	15 (15.3)	13 (13.3)	18 (18.4)	23 (23.5)	26 (26.5)	< 5 (−)
	Classification method 3							
	*HER2+*	61	15 (24.6)	12 (19.7)	18 (29.5)	< 5 (−)	11 (18.0)	< 5 (−)
	*Not-HER2+ or Unknown*	228	< 5 (−)	< 5 (−)	< 5 (−)	77 (33.8)	133 (58.3)	15 (6.6)
**Further lines**	Classification method 1							
	*Luminal A*	125	< 5 (−)	< 5 (−)	< 5 (−)	66 (52.8)	57 (45.6)	< 5 (−)
	*Luminal B*	363	20 (5.5)	17 (4.7)	52 (14.3)	140 (38.6)	120 (33.1)	14 (3.9)
	*Triple-negative*	18	< 5 (−)	< 5 (−)	< 5 (−)	17 (94.4)	< 5 (−)	< 5 (−)
	*HR-/HER2+*	18	< 5 (−)	< 5 (−)	11 (61.1)	< 5 (−)	< 5 (−)	< 5 (−)
	*Unknown*	78	< 5 (−)	< 5 (−)	< 5 (−)	46 (59.0)	30 (38.5)	< 5 (−)
	Classification method 2							
	*HR+/HER2−*	408	< 5 (−)	< 5 (−)	< 5 (−)	217 (53.2)	176 (43.1)	12 (2.9)
	*Not-HR+/HER2− or unknown*	194	23 (11.9)	16 (8.2)	65 (33.5)	55 (28.4)	31 (16.0)	< 5 (−)
	Classification method 3							
	*HER2+ *	121	22 (18.2)	16 (13.2)	63 (52.1)	6 (5.0)	10 (8.3)	< 5 (−)
	*Not-HER2+ or unknown*	481	< 5 (−)	< 5 (−)	< 5 (−)	266 (55.3)	197 (41.0)	12 (2.5)

*aBC* advanced breast cancer, *HER2* human epidermal growth factor receptor 2, *HR* hormone receptor

### Overall and metastasis-free survival

**Fig. 1:**
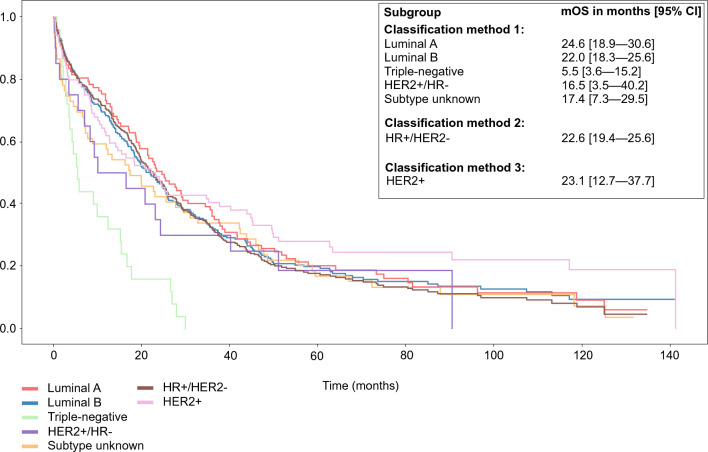
Overall survival (OS) from aBC diagnosis in different breast cancer subgroups. *aBC* advanced breast cancer, *CI* confidence interval, *HER2* human epidermal growth factor receptor 2, *HR* hormone receptor, *mOS* median overall survival

The mOS of all aBC patients was 20.1 months (95% confidence interval, CI 17.7–23.4). Within classification 1, the longest mOS was observed in the luminal A subgroup (24.6 months; CI 18.9–30.6) and the shortest in the triple-negative subgroup (5.5 months; CI 3.6–15.2) (Fig. 1). Neither luminal A nor B mOS confidence intervals overlapped with that of the triple-negative subgroup. For the HR+/HER2− patients (classification 2), the mOS was 22.6 months (CI 19.4–25.6) and for the HER2+ patients (classification 3) 23.1 months (CI 12.7–37.7).

**Fig. 2:**
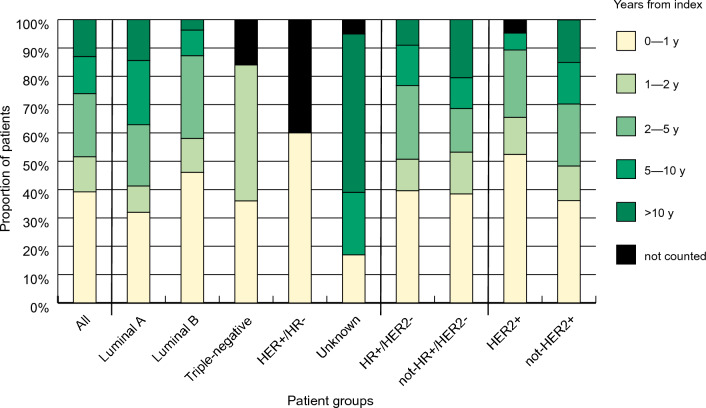
Metastasis-free survival (MFS). Proportions of patients diagnosed with metastasis at different time intervals from the index date. Groups of less than 5 patients were not included in the analysis. *y* year. *HER2* human epidermal growth factor receptor 2, *HR* hormone receptor

Over one-third of all patients (39.2%) developed metastasis within one year and 13.1% after 10 years from the initial diagnosis (Fig. 2; Supplementary Table 3). When examining MFS by subgroups, in classification 1 the HR−/HER2+ subgroup had the largest proportion of patients (60.0%) who developed metastasis during the first year after being diagnosed with BC. However, it should be noted that the group size is small (*n* = 20). In classifications 2 and 3, 39.6% of the patients in the HR+ /HER2− subgroup and 52.4% of the HER2+ patients developed metastasis during the first year. A majority (84.0%) of the triple-negative patients developed metastasis during the first two years after the diagnosis, whereas in the other subgroups the development of metastasis was more evenly dispersed over a longer time period.

### PIK3CA mutations

A total of 187 patients with HR+/HER2− aBC were identified for *PIK3CA* mutation analysis. Of these, *PIK3CA* was sequenced from 161 (86.1%) tumors, excluding 26 (13.9%) samples due to low DNA concentration. Out of the 161 HR+/HER2− tumors, 53.4% harbored a *PIK3CA* mutation and 46.6% were wt (Table 4). *PIK3CA* hotspot mutation was found in 32.3% of all the screened tumors.

**Table 4:** *PIK3CA* mutation subgroups, number of cases, and median OS

Subgroup	Patients *n* (% of all screened HR+/HER2− patients)	Median OS in months [95% CI]
No mutation	75 (46.6%)	18.9 [14.1–25.1]
Hotspot mutation	52 (32.3%)	22.6 [17.0–26.3]
Other mutation	34 (21.1%)	23.4 [8.2–36.5]
Any mutation	86 (53.4%)	23.4 [17.0–27.6]

*CI* confidence interval, *HER2* human epidermal growth factor receptor 2, *HR* hormone receptor, *OS* overall survival

The most common hotspot mutations were H1047R (26 tumors), E542K (10), and E545K (8) (Supplementary Table 4). Outside the mutation hotspots, the most common mutation was N345K, which was found in six tumors. Out of the 86 tumors with a *PIK3CA* mutation, 29 (33.7%) displayed more than one *PIK3CA* mutation, i.e., mostly two or three different variants.

Patients with an HR+/HER2−, *PIK3CA* wt tumor appeared to have shorter mOS, 18.9 months (CI 14.1–25.1), compared to patients with a tumor harboring any *PIK3CA* mutation (23.4 months; CI 17.0–27.6) (Table 4).

## Discussion

Although BC treatment has significantly improved and mortality has declined during the last decades in most developed countries, it remains one of the leading causes of cancer deaths worldwide (Sung et al. 2021). Due to advances in cancer research during the past decade, tumors can be classified more specifically into different subtypes. However, more data are needed to understand their distribution and outcomes in a real-life clinical setting. In this study, we report both general and subtype-level information on aBC from real-life clinical practice in Finland.

Altogether, 444 aBC patients diagnosed in the Hospital District of Southwest Finland between 2004 and 2013 were included in the study. During the study period, the percentage of new aBC diagnoses out of all new BC cases showed a steady increase until 2010, after which it remained stable. This is in line with the reported BC incidence of both Finland and the Western countries in general, where the incidence of BC showed a small to moderate increase (Sung et al. 2021; Pitkäniemi et al. 2019). The incidence of de novo metastatic BC has been reported to be stable (Malmgren et al. 2018; Valachis et al. 2021).

In this study, patients were classified into different BC subtypes based on tumor marker profiles using three different classification methods. The proportion of triple-negative patients was lower than reported earlier (5.6% vs. 15–20%), explained partly due to using ≥ 1% as threshold for ER/PR positivity. The proportion of HER2+ patients (18.9%) was similar to a previous Finnish study cohort (19%) (Yao et al. 2016; Joensuu et al. 2017; Łukasiewicz et al. 2021). Also, the proportion of luminal cancers (76.5%) corresponded to a previously reported fraction of around 70% (Łukasiewicz et al. 2021).

Much of the published aBC outcome data derive from clinical trials with selected patient populations. Real-world data on subtype-specific aBC survival in Finland has been scarce. In general, the mOS of aBC patients is approximately three years (Mattson and Huovinen 2015). In our study, the mOS was clearly shorter (20 months), regardless of the lower proportion of triple-negative cancers than generally reported (Yao et al. 2016; Łukasiewicz et al. 2021). It is worth noting that 12.8% of patients in our study cohort (57/444) did not receive any pharmacological treatment, which affects the mOS considerably. The survival analysis also revealed variation between the different aBC subtypes. In classification 1, the triple-negative BC patients had a shorter mOS (5.5 months) compared to other patient subgroups (16.5–24.6 months), which is in line with previous literature (Sporikova et al. 2018). Triple-negative BC patients have also been shown to have markedly worse survival among early BC patients in another Finnish study (Teerenhovi et al. 2021). In this study, the HR+/HER2− and HER2+ patients showed similar mOS (22.6 and 23.1 months, respectively) supporting the notion that in the era of HER2-targeted therapy, the prognostic relevance of HER2-positivity has shifted toward having a more favorable influence on patient survival (Lobbezoo et al. 2013).

More than one-third of the aBC patients developed metastasis within a year from the initial BC diagnosis. In the triple-negative subgroup, most patients developed metastasis within two years after diagnosis, while in other subgroups, the development of metastasis was more evenly distributed over a longer timeframe. This pattern is similar to what has previously been observed and might reflect the aggressive nature of triple-negative BC and lack of available effective treatments specific for this subtype (Bauer et al. 2007; Dent et al. 2007).

Most patients had received at least one line of therapy. The percentage of patients receiving therapy was lower in triple-negative and HR−/HER2+ groups in all lines compared to luminal A and B. Differences in the number of patients receiving treatment could possibly be contributed by ineligibility for treatments due to health issues, variation of available treatments for specific subtypes, and the fact that patients with triple-negative BC are less likely to receive multiple lines of treatment due to their shorter mOS.

Some variation also existed between the type of received treatment within and between subgroups. Variation between subgroups can be explained mostly by the specific treatments targeted for certain subtypes. The triple-negative cancers were treated with only chemotherapy in the first line, as recommended by the treatment guidelines (Suomen Rintasyöpäryhmä RY 2023). The majority (68.1%) of the patients with HER2+ cancer (classification 3) had received the recommended combination of anti-HER2 and chemotherapy as a first-line treatment. The number of patients with luminal cancer receiving only chemotherapy as first-line therapy was relatively high, which is due to the fact that only recently has first-line treatment shifted toward hormonal therapy and CDK4/6 inhibitors, which were not available during the study period. Luminal A cancers were treated mainly (56.8%) with only endocrine therapy; however, a large number of patients (40.9%) had received only chemotherapy. For luminal B cancers, chemotherapy only was the most common (40.6%) first-line therapy. This was followed by endocrine only and anti-HER2+ chemotherapy. Variation of treatments within subgroups, and in further therapy lines, may reflect, e.g., the heterogeneity of the patients, their wishes for the treatment type, their previously received treatments, and the availability of different treatments during the study period. This study did not capture the use of radiotherapy, which is likely to have and influence on patient’s overall treatment scheme and survival.

Around one-third of the sequenced HR+/HER2− cancers harbored a *PIK3CA* hotspot mutation which is in line with previously reported results (The Cancer Genome Atlas Network 2012; Moynahan et al. 2017; Di Leo et al. 2018; Hortobagyi et al. 2018; André et al. 2019). In this study, patients with *PIK3CA* wt tumor had a shorter mOS than patients having *PIK3CA*-mutated aBC. In general, multiple studies have shown *PIK3CA* as a negative prognostic factor (Li et al. 2006; Lai et al. 2008; Aleskandarany et al. 2010; Sobhani et al. 2018; Mosele et al. 2020). Given the relatively low number of patients in this study, and the fact that the study did not account for other driver mutations, clinicopathological features (such as tumor stage, size, or histological grade), or the treatment, the results should be interpreted with caution. However, HR+/HER2− BCs with *PIK3CA* mutations are associated with chemoresistance and thus these patients represent a subgroup with an unmet medical need (Mosele et al. 2020). This patient population could benefit from PI3K inhibitors, such as alpelisib, that have been shown to improve PFS significantly and mOS numerically (André et al. 2019, 2021).

As register-based studies in general, this study may have limitations associated with the completeness and accuracy of the data. Even if the validation of the study population was done in several steps, it is still possible that not all aBC patients were captured. In addition, mutation screening was impossible for some samples due to poor DNA quality, and there was a small sample size in certain patient subgroups (HR−/HER2+ and triple-negative). Thus, the results of these subgroups need to be interpreted with caution. It should also be noted that although all aBC patients meeting the inclusion criteria were included in the study, the overall sample size of the study is limited and covers only one hospital district, and thus generalization of the results on a national level should also be done with caution. However, as all identified aBC patients were included in the study, there was no selection bias regarding the study population, and this cohort may represent the general population rather well. Overall, this study provides valuable information on aBC patients in Finland, the distribution of different BC subtypes, and their clinical outcomes.

## Conclusion

To our knowledge, this is among the first retrospective register-based studies reporting overall and metastasis-free survival among different aBC patient subgroups in Finland. The data presented here are consistent with previous data for the triple-negative BC patients showing the aggressive nature of the disease. Most triple-negative patients recur within the first two years from the primary diagnosis in contrast to the other subgroups. In addition, HR+/HER2− and HER2+ patients showed similar mOS outcomes, while triple-negative patients demonstrated shorter mOS. Previously, real-life data on subtype-specific *PIK3CA* mutation fractions has largely been lacking. Here, one-third of the sequenced cancers showed a *PIK3CA* hotspot mutation. Although these mutations did not lead to worse survival in this study, they are relevant as possible treatment targets for this patient population. Overall, this type of data can be utilized for further evaluation of the medical need in different breast cancer subgroups.

## Supplementary Information

Below is the link to the electronic supplementary material.

Supplementary file1 (DOCX 62 KB)

## Data Availability

The datasets generated and/or analyzed during the current study are available from the corresponding author on reasonable request.

## Abbreviations

aBC: Advanced breast cancer
BC: Breast cancer
CDK4/6: Cyclin-dependent kinase 4 and 6
CI: Confidence interval
EMR: Electronic medical records
ER: Estrogen receptor
ESMO: European society for medical oncology
FFPE: Formalin-fixed, paraffin-embedded
HER2: Human epidermal growth factor receptor 2
HR: Hormone receptor
MFS: Metastasis-free survival
mOS: Median overall survival
NGS: Next generation sequencing
OS: Overall survival
PIK3CA: Phosphatidylinositol-4,5-bisphosphate 3-kinase catalytic subunit alpha
PR: Progesterone receptor
SD: Standard deviation
TP53: Tumor protein P53
wt: Wild-type

## References

[CR1] Aleskandarany MA, Rakha EA, Ahmed MAH, Powe DG, Paish EC, Macmillan RD, Ellis IO, Green AR (2010). PIK3CA expression in invasive breast cancer: a biomarker of poor prognosis. Breast Cancer Res Treat.

[CR2] André F, Ciruelos E, Rubovszky G, Campone M, Loibl S, Rugo HS, Iwata H, Conte P, Mayer IA, Kaufman B, Yamashita T, Lu Y-S, Inoue K, Takahashi M, Pápai Z, Longin A-S, Mills D, Wilke C, Hirawat S, Juric D (2019). Alpelisib for PIK3CA-mutated, hormone receptor-positive advanced breast cancer. New Engl J Med..

[CR3] André F, Ciruelos EM, Juric D, Loibl S, Campone M, Mayer IA, Rubovszky G, Yamashita T, Kaufman B, Lu Y-S, Inoue K, Pápai Z, Takahashi M, Ghaznawi F, Mills D, Kaper M, Miller M, Conte PF, Iwata H, Rugo HS (2021). Alpelisib plus fulvestrant for PIK3CA-mutated, hormone receptor-positive, human epidermal growth factor receptor-2–negative advanced breast cancer: final overall survival results from SOLAR-1. Ann Oncol.

[CR4] Bauer KR, Brown M, Cress RD, Parise CA, Caggiano V (2007). Descriptive analysis of estrogen receptor (ER)-negative, progesterone receptor (PR)-negative, and HER2-negative invasive breast cancer, the so-called triple-negative phenotype. Cancer.

[CR5] Cardoso F, Paluch-Shimon S, Senkus E, Curigliano G, Aapro MS, André F, Barrios CH, Bergh J, Bhattacharyya GS, Biganzoli L, Boyle F, Cardoso M-J, Carey LA, Cortés J, El Saghir NS, Elzayat M, Eniu A, Fallowfield L, Francis PA, Gelmon K, Gligorov J, Haidinger R, Harbeck N, Hu X, Kaufman B, Kaur R, Kiely BE, Kim S-B, Lin NU, Mertz SA, Neciosup S, Offersen BV, Ohno S, Pagani O, Prat A, Penault-Llorca F, Rugo HS, Sledge GW, Thomssen C, Vorobiof DA, Wiseman T, Xu B, Norton L, Costa A, Winer EP (2020). 5th ESO-ESMO international consensus guidelines for advanced breast cancer (ABC 5). Ann Oncol.

[CR6] Dent R, Trudeau M, Pritchard KI, Hanna WM, Kahn HK, Sawka CA, Lickley LA, Rawlinson E, Sun P, Narod SA (2007). Triple-negative breast cancer: clinical features and patterns of recurrence. Clin Cancer Res.

[CR7] Di Leo A, Johnston S, Lee KS, Ciruelos E, Lønning PE, Janni W, O’Regan R, Mouret-Reynier M-A, Kalev D, Egle D, Csőszi T, Bordonaro R, Decker T, Tjan-Heijnen VCG, Blau S, Schirone A, Weber D, El-Hashimy M, Dharan B, Sellami D, Bachelot T (2018). Buparlisib plus fulvestrant in postmenopausal women with hormone-receptor-positive, HER2-negative, advanced breast cancer progressing on or after mTOR inhibition (BELLE-3): a randomised, double-blind, placebo-controlled, phase 3 trial. Lancet Oncol.

[CR8] Gennari A, André F, Barrios CH, Cortés J, de Azambuja E, DeMichele A, Dent R, Fenlon D, Gligorov J, Hurvitz SA, Im S-A, Krug D, Kunz WG, Loi S, Penault-Llorca F, Ricke J, Robson M, Rugo HS, Saura C, Schmid P, Singer CF, Spanic T, Tolaney SM, Turner NC, Curigliano G, Loibl S, Paluch-Shimon S, Harbeck N (2021). ESMO clinical practice guideline for the diagnosis, staging and treatment of patients with metastatic breast cancer. Ann Oncol.

[CR9] Hortobagyi GN, Stemmer SM, Burris HA, Yap YS, Sonke GS, Paluch-Shimon S, Campone M, Petrakova K, Blackwell KL, Winer EP, Janni W, Verma S, Conte P, Arteaga CL, Cameron DA, Mondal S, Su F, Miller M, Elmeliegy M, Germa C, O’Shaughnessy J (2018). Updated results from MONALEESA-2, a phase III trial of first-line ribociclib plus letrozole versus placebo plus letrozole in hormone receptor-positive, HER2-negative advanced breast cancer. Ann Oncol.

[CR10] Joensuu H, Kellokumpu-Lehtinen P-L, Huovinen R, Jukkola-Vuorinen A, Tanner M, Kokko R, Ahlgren J, Auvinen P, Lahdenperä O, Kosonen S, Villman K, Nyandoto P, Nilsson G, Poikonen-Saksela P, Kataja V, Junnila J, Bono P, Lindman H (2017). Adjuvant capecitabine in combination with docetaxel, epirubicin, and cyclophosphamide for early breast cancer: the randomized clinical FinXX trial. JAMA Oncol.

[CR11] Lai Y-L, Mau B-L, Cheng W-H, Chen H-M, Chiu H-H, Tzen C-Y (2008). PIK3CA Exon 20 mutation is independently associated with a poor prognosis in breast cancer patients. Ann Surg Oncol.

[CR12] Li SY, Rong M, Grieu F, Iacopetta B (2006). PIK3CA mutations in breast cancer are associated with poor outcome. Breast Cancer Res Treat.

[CR13] Lobbezoo DJA, van Kampen RJW, Voogd AC, Dercksen MW, van den Berkmortel F, Smilde TJ, van de Wouw AJ, Peters FPJ, van Riel JMGH, Peters NAJB, de Boer M, Borm GF, Tjan-Heijnen VCG (2013). Prognosis of metastatic breast cancer subtypes: the hormone receptor/HER2-positive subtype is associated with the most favorable outcome. Breast Cancer Res Treat.

[CR14] Łukasiewicz S, Czeczelewski M, Forma A, Baj J, Sitarz R, Stanisławek A (2021). Breast cancer—epidemiology, risk factors, classification, prognostic markers, and current treatment strategies—an updated review. Cancers.

[CR15] Malmgren JA, Mayer M, Atwood MK, Kaplan HG (2018). Differential presentation and survival of de novo and recurrent metastatic breast cancer over time: 1990–2010. Breast Cancer Res Treat.

[CR16] Mattson J, Huovinen R (2015). Levinneen Rintasyövän Hoito. LÄÄKETIETEELLINEN AIKAKAUSKIRJA DUODECIM.

[CR17] Mosele F, Stefanovska B, Lusque A, Tran DA, Garberis I, Droin N, Le Tourneau C, Sablin M-P, Lacroix L, Enrico D, Miran I, Jovelet C, Bièche I, Soria J-C, Bertucci F, Bonnefoi H, Campone M, Dalenc F, Bachelot T, Jacquet A, Jimenez M, André F (2020). Outcome and molecular landscape of patients with PIK3CA-mutated metastatic breast cancer. Ann Oncol.

[CR18] Moynahan ME, Chen D, He W, Sung P, Samoila A, You D, Bhatt T, Patel P, Ringeisen F, Hortobagyi GN, Baselga J, Chandarlapaty S (2017). Correlation between PIK3CA mutations in cell-free DNA and everolimus efficacy in HR+, HER2− advanced breast cancer: results from BOLERO-2. Br J Cancer.

[CR19] Pitkäniemi J, Malila N, Tanskanen T, Degerlund H, Heikkinen S, Seppä K Syöpä (2019) Tilanneraportti Suomen syöpätilanteesta. https://syoparekisteri.fi/assets/files/2021/05/Syopa_2019_tilastoraportti.pdf. Accessed 5 May 2023

[CR20] Sobhani N, Roviello G, Corona SP, Scaltriti M, Ianza A, Bortul M, Zanconati F, Generali D (2018). The prognostic value of PI3K mutational status in breast cancer: a meta-analysis. J Cell Biochem.

[CR21] Sporikova Z, Koudelakova V, Trojanec R, Hajduch M (2018). Genetic markers in triple-negative breast cancer. Clin Breast Cancer.

[CR22] Sung H, Ferlay J, Siegel RL, Laversanne M, Soerjomataram I, Jemal A, Bray F (2021). Global cancer statistics 2020: GLOBOCAN estimates of incidence and mortality worldwide for 36 cancers in 185 countries. CA Cancer J Clin.

[CR23] Suomen Rintasyöpäryhmä RY Rintasyövän valtakunnallinen diagnostiikka- ja hoitosuositus. https://rintasyoparyhma.yhdistysavain.fi/hoitosuositus/

[CR24] Teerenhovi H, Tuominen S, Nurmi-Rantala S, Hemmilä P, Ellonen A (2021). Real-world clinical outcomes in biological subgroups of breast cancer in the hospital district of southwest Finland. Oncologist.

[CR25] The Cancer Genome Atlas Network (2012). Comprehensive molecular portraits of human breast tumours. Nature.

[CR26] Valachis A, Carlqvist P, Szilcz M, Freilich J, Vertuani S, Holm B, Lindman H (2021). Use of classifiers to optimise the identification and characterisation of metastatic breast cancer in a nationwide administrative registry. Acta Oncol.

[CR27] Yao H, He G, Yan S, Chen C, Song L, Rosol TJ, Deng X (2016). Triple-negative breast cancer: is there a treatment on the horizon?. Oncotarget.

[CR28] Yersal O, Barutca S (2014). Biological subtypes of breast cancer: prognostic and therapeutic implications. World J Clin Oncol.

