# Supplementary material for: Characterization of clinicopathological features, treatment practices, and outcomes among Finnish advanced breast cancer patients in real-life clinical practice
Source: J Cancer Res Clin Oncol. 2023 May 13;149(11):9139–49. doi: 10.1007/s00432-023-04723-0 (PMC10374819; doi:10.1007/s00432-023-04723-0)
Supplement: Supplementary file 1 — Supplementary file1 (DOCX 62 KB) [file 432_2023_4723_MOESM1_ESM.docx]

**Supplementary Information**

**Characterization of clinicopathological features, treatment practices, and outcomes among Finnish advanced breast cancer patients in real-life clinical practice**

**Journal:** Breast Cancer Research and Treatment

Krista Heinolainen (1), Silva Saarinen (1), Simona Vertuani (2), Antti Ellonen (3), Antti Karlsson (4), Meri Utriainen (5), Peter Carlqvist (6), Jami Mandelin (7), Barbro Holm (2)

1. Novartis Finland Oy, Espoo, Finland
2. Novartis Sverige AB, Kista, Sweden
3. Department of Oncology, Turku University Central Hospital, Turku, Finland
4. Auria Biobank and University of Turku, Turku, Finland
5. Comprehensive Cancer Center, Helsinki University Central Hospital, Helsinki, Finland
6. Nordic Market Access, Stockholm, Sweden
7. Faron Pharmaceuticals, Turku, Finland

Email (and ORCID id) of the corresponding author:

Email: [barbro.holm@novartis.com](mailto:barbro.holm@novartis.com)

ORCID ID: 0000-0002-2944-9464

**Supplementary Table 1**. Distribution of treatment types in the first, second, and further therapy lines. Groups of less than five patients were not included in the analysis. HER2, human epidermal growth factor receptor 2.

| Therapy line | No. of observed lines | Anti-HER2 therapy  n (%) | Anti-HER2+ endocrine  n (%) | Anti-HER2+ chemo-therapy  n (%) | Chemo-therapy only  n (%) | Endocrine only  n (%) | Other treatment  n (%) |
| --- | --- | --- | --- | --- | --- | --- | --- |
| First line | 387 | <5 (-) | <5 (-) | 51 (13.2) | 158 (40.8) | 161 (41.6) | 12 (3.1) |
| Second line | 289 | 16 (5.5) | 13 (4.5) | 19 (6.6) | 79 (27.3) | 144 (49.8) | 18 (6.2) |
| Later lines | 602 | 25 (4.2) | 17 (2.8) | 65 (10.8) | 272 (45.2) | 207 (34.4) | 16 (2.7) |

**Supplementary Table 2**. Treated patients by subgroup and treatment line. A summary of the therapy lines and distribution of patient subgroups. HER2, human epidermal growth factor receptor 2; HR, hormone receptor.

| Subgroup | n | First-line therapy | Second-line therapy | Further therapy lines |
| --- | --- | --- | --- | --- |
| **Classification 1:** | | | | |
| Luminal A | 97 | 88 (90.7%) | 65 (67.0%) | 48 (49.5%) |
| Luminal B | 243 | 219 (90.1%) | 166 (68.3%) | 119 (49.0%) |
| Triple-negative | 25 | 18 (72.0%) | 11 (44.0%) | 7 (28.0%) |
| HR-/HER2+ | 20 | 15 (75.0 %) | 12 (60.0%) | 6 (30.0%) |
| unknown | 59 | 47 (79.7%) | 35 (59.3%) | 24 (40.7%) |
| **Classification 2:** | | | | |
| HR+/HER2- | 288 | 260 (90.3%) | 191 (66.3%) | 141 (49.0%) |
| Not-HR+/HER2- or unknown | 156 | 127 (81.4%) | 98 (62.8%) | 63 (40.4%) |
| **Classification 3:** | | | | |
| HER2+ | 84 | 72 (85.7%) | 61 (72.6%) | 39 (46.4%) |
| Not-HER2+ or unknown | 360 | 315 (87.5%) | 228 (63.3%) | 165 (45.8%) |

**Supplementary Table 3.** Overall metastasis-free survival, among all and by subgroups. Groups of less than five people were not included in the analysis. HER2, human epidermal growth factor receptor 2; HR, hormone receptor.

| Subgroup | Time to metastasis (range, in years) | Patients (N) | Percentage (%) |
| --- | --- | --- | --- |
| **All** | 0–1 | 174 | 39.2 |
|  | 1–2 | 55 | 12.4 |
|  | 2–5 | 99 | 22.3 |
|  | 5–10 | 58 | 13.1 |
|  | >10 | 58 | 13.1 |
| **Classification method 1:** | | | |
| **Luminal A** | 0–1 | 31 | 32.0 |
|  | 1–2 | 9 | 9.3 |
|  | 2–5 | 21 | 21.6 |
|  | 5–10 | 22 | 22.7 |
|  | >10 | 14 | 14.4 |
| **Luminal B** | 0–1 | 112 | 46.1 |
|  | 1–2 | 29 | 11.9 |
|  | 2–5 | 71 | 29.2 |
|  | 5–10 | 22 | 9.1 |
|  | >10 | 9 | 3.7 |
| **Triple-negative** | 0–1 | 9 | 36.0 |
|  | 1–2 | 12 | 48.0 |
|  | 2–5 | <5 | - |
|  | 5–10 | <5 | - |
|  | >10 | <5 | - |
| **HR-/HER2+** | 0–1 | 12 | 60.0 |
|  | 1–2 | <5 | - |
|  | 2–5 | <5 | - |
|  | 5–10 | <5 | - |
|  | >10 | <5 | - |
| **unknown** | 0–1 | 10 | 16.9 |
|  | 1–2 | <5 | - |
|  | 2–5 | <5 | - |
|  | 5–10 | 13 | 22.0 |
|  | >10 | 33 | 55.9 |
| **Classification method 2:** | | | |
| **HR+/HER2-** | 0–1 | 114 | 39.6 |
|  | 1–2 | 32 | 11.1 |
|  | 2–5 | 75 | 26.0 |
|  | 5–10 | 41 | 14.2 |
|  | >10 | 26 | 9.0 |
| **Not-HR+/HER2- or unknown** | 0–1 | 60 | 38.5 |
|  | 1–2 | 23 | 14.7 |
|  | 2–5 | 24 | 15.4 |
|  | 5–10 | 17 | 10.9 |
|  | >10 | 32 | 20.5 |
| **Classification method 3:** | | | |
| **HER2+** | 0–1 | 44 | 52.4 |
|  | 1–2 | 11 | 13.1 |
|  | 2–5 | 20 | 23.8 |
|  | 5–10 | 5 | 6.0 |
|  | >10 | <5 | - |
| **Not-HER2+ or unknown** | 0–1 | 130 | 36.1 |
|  | 1–2 | 44 | 12.2 |
|  | 2–5 | 79 | 21.9 |
|  | 5–10 | 53 | 14.7 |
|  | >10 | 54 | 15.0 |

**Supplementary Table 4**. The numbers of different *PIK3CA* hotspot mutations found in the sequenced HR+/HER2- aBCs (n=161).

| *PIK3CA* hotspot mutations | Total number of mutations |
| --- | --- |
| C420R | 2 |
| E542K | 10 |
| E545A | 1 |
| E545K | 8 |
| H1047L | 5 |
| H1047R | 26 |
| H1047Y | 2 |
| Q546K | 1 |
